# Systematic Analysis of Head-to-Head Gene Organization: Evolutionary Conservation and Potential Biological Relevance

**DOI:** 10.1371/journal.pcbi.0020074

**Published:** 2006-07-07

**Authors:** Yuan-Yuan Li, Hui Yu, Zong-Ming Guo, Ting-Qing Guo, Kang Tu, Yi-Xue Li

**Affiliations:** 1 Shanghai Center for Bioinformation Technology, Shanghai, People's Republic of China; 2 Institute of Biochemistry and Cell Biology, Shanghai Institutes for Biological Sciences, Chinese Academy of Sciences, Shanghai, People's Republic of China; 3 Bioinformatics Center, Shanghai Institutes for Biological Sciences, Chinese Academy of Sciences, Shanghai, People's Republic of China; Memorial Sloan-Kettering Cancer Center, United States of America

## Abstract

Several “head-to-head” (or “bidirectional”) gene pairs have been studied in individual experiments, but genome-wide analysis of this gene organization, especially in terms of transcriptional correlation and functional association, is still insufficient. We conducted a systematic investigation of head-to-head gene organization focusing on structural features, evolutionary conservation, expression correlation and functional association. Of the present 1,262, 1,071, and 491 head-to-head pairs identified in human, mouse, and rat genomes, respectively, pairs with 1– to 400–base pair distance between transcription start sites form the majority (62.36%, 64.15%, and 55.19% for human, mouse, and rat, respectively) of each dataset, and the largest group is always the one with a transcription start site distance of 101 to 200 base pairs. The phylogenetic analysis among *Fugu,* chicken, and human indicates a negative selection on the separation of head-to-head genes across vertebrate evolution, and thus the ancestral existence of this gene organization. The expression analysis shows that most of the human head-to-head genes are significantly correlated, and the correlation could be positive, negative, or alternative depending on the experimental conditions. Finally, head-to-head genes statistically tend to perform similar functions, and gene pairs associated with the significant cofunctions seem to have stronger expression correlations. The findings indicate that the head-to-head gene organization is ancient and conserved, which subjects functionally related genes to correlated transcriptional regulation and thus provides an exquisite mechanism of transcriptional regulation based on gene organization. These results have significantly expanded the knowledge about head-to-head gene organization. Supplementary materials for this study are available at http://www.scbit.org/h2h.

## Synopsis

It was commonly assumed that higher eukaryotic genomes are loosely organized and genes are interspersed in the whole genome sequences. However, experiments have continuously identified eukaryotic head-to-head gene pairs with genes located closely next to each other, possibly sharing a same promoter; and preliminary genomic surveys have even proved head-to-head gene pair to be a common feature of human genome. The authors report a systematic investigation of head-to-head gene pairs in terms of the genomic structure, evolutionary conservation, expressional correlation, and functional association. The authors first identified some common structural and distributional patterns in three representative mammalian genomes: human, mouse, and rat. Then, through comparative analyses between human, chicken, and *Fugu,* they observed a conservation tendency of head-to-head gene pairs in vertebrates. Finally, interactive analyses of expressional and functional association yielded some interesting results, including the significant expression correlation of head-to-head genes, especially for the pairs with significant functional association. The main conclusion of this paper is that the head-to-head gene organization is ancient and conserved, subjecting functionally related genes to coregulated transcription. Lists of head-to-head gene pairs in human, mouse, rat, chicken, and *Fugu* are provided, while some individual pairs in need of further in-depth investigations are highlighted.

## Introduction

**Figure 1:**
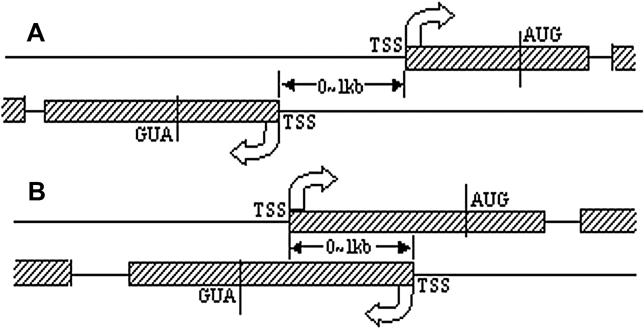
A Schematic Illustration of Head-to-Head Gene Organization (A and B) Nonoverlapping and overlapping head-to-head gene pairs, respectively.

A “head-to-head” or “bidirectional” gene pair describes a genomic locus in which two adjacent genes are divergently transcribed from opposite strands of DNA, and the region between two transcription start sites (TSSs) is commonly designated as a putative bidirectional promoter [1, 2] (see Figure 1 for the definition of head-to-head gene organization). This gene organization was first observed in the investigation of mouse *DHFR* gene [3]. Subsequently, *SURF-1*/*SURF-2* [4], *COL4A1*/*COL4A2* [1], *RanBP1*/*Htf9-c* [5], *E14*/*ATM* [6], *BRCA1*/*NBR2* [7], *DNA-PKcs*/*MCM4* [8], *FEN1*/*C11orf10* [9], and so on were identified in human, hamster, rat, or mouse through individual experiments. Of them, many cases, such as *DHFR*/*REP3* [10], *SURF-1*/*SURF-2* [11], *E14*/*ATM* [6], and *TK*/*KF* [12], were found to be conserved among mammalian species. Computational analysis revealed that more than 10% of human genes were organized in this head-to-head manner separated by less than 1,000 base pairs (bp), suggesting that bidirectional gene organization seems to be a common architectural feature of the human genome [2, 9].

Examination of individual examples showed that a bidirectional promoter tends to coordinately regulate the transcription of the involved gene pair. Some head-to-head genes are positively correlated and function in the same pathway, such as human collagen genes *COL4A1*/*COL4A2* [1, 13] and chicken genes *GPAT*/*AIRC* involved in de novo purine nucleotide synthesis [14]; some are coregulated in a common window of the cell cycle, such as murine genes *RanBP1*/*Htf9-c* [5, 15]; some are coordinated to respond to induction signals, for example, human genes *HSP60*/*HSP10* [16]. However, there are also some rare examples of negatively correlated head-to-head genes, such as mouse genes *TK*/*KF* [12]. Given that head-to-head gene organization has been found to be a common architectural feature [2], it is necessary to reevaluate the underlying mechanisms and biological relevance systematically.

In this paper, we performed genome-wide identification of head-to-head gene pairs in human, mouse, and rat genomes and analyzed structural features of this gene organization in mammalian genomes. Then we studied the conservation of the gene arrangement during vertebrate evolution using human, chicken, and *Fugu* genomic data. Furthermore, we examined the expression correlation and functional association between human head-to-head genes. Our results suggest that the conserved head-to-head gene organization provides a unique mechanism of transcriptional regulation for functionally related genes in vertebrates.

## Results

### Identification and Characterization of Head-to-Head Gene Pairs in Human, Mouse, and Rat Genome

A total of 1,262 human head-to-head gene pairs with their TSSs separated by less than 1 kb were identified from 26,813 human genes according to the genomic mapping data from the National Center for Biotechnology Information (NCBI) (see Table S1, “H2Hpairs” sheet, for detailed information of each pair). The mitochondrial genome was ignored in this work since its organization is far more compact than that of the nuclear genome. Given a situation that one gene could be covered by two pairs simultaneously due to a close arrangement of two genes (Table S1, “GenesInMultiH2H” sheet), the 1,262 pairs involve a total of 2,515 genes. That is, 9.4% of human genes are organized in a head-to-head configuration. Similarly, 1,071 and 491 head-to-head pairs, corresponding to 2,130 (8.2%) and 968 (4.4%) genes, were identified from 25,841 mouse genes and 21,977 rat genes, respectively (see Tables S2 and S3 for detailed information).

**Figure 2:**
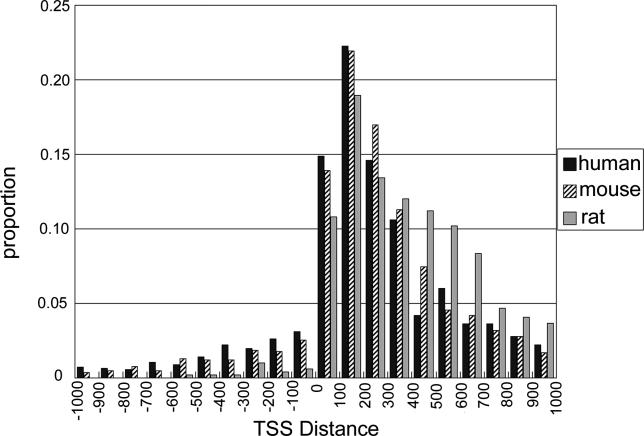
Distribution of TSS Distance of Head-to-Head Genes

To characterize structural features of head-to-head gene organization in mammalian genomes, we determined the distributions of TSS distance of the human, mouse, and rat head-to-head gene pairs. The three species show similar distribution plots (Figure 2), where four columns representing pairs with TSS distance of 1 to 400 bp contain the majority (62.36%, 64.15%, and 55.19% for human, mouse, and rat, respectively) of the total number of pairs, and the peak is always the group with 101- to 200-bp distance (see Table S1, “DistHist” sheet for detailed data). The obviously lower number of rat head-to-head pairs and their relatively flat profile of the distance distribution might be attributed to the incomplete 5' UTR information and thus the imprecise calculation of TSS distances, which will be further explained in the Discussion section.

**Figure 3:**
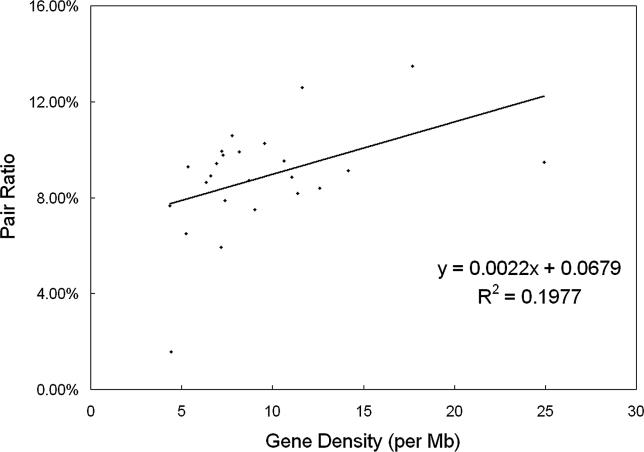
Relationship between Head-to-Head Gene Pair Ratio and Gene Density

All head-to-head gene pairs identified in this paper were mapped to the whole human genome (Figure S1). Also, the relationship between head-to-head pair ratios and gene densities of each chromosome was examined statistically (Table 1). The pair ratio was obtained by dividing the number of genes involved in head-to-head pairs (h2h gene number) by the total gene count in a certain chromosome. The Pearson correlation coefficient indicates that there is a significant linear relationship between pair ratio and gene density at *p* < 0.05 (Figure 3), contradicting the previous report based on the data from Chromosomes 21 and 22 [9]. A significant linear relationship was also observed in mouse genome (see Table S2, “DistHist” sheet).

**Table 1:**
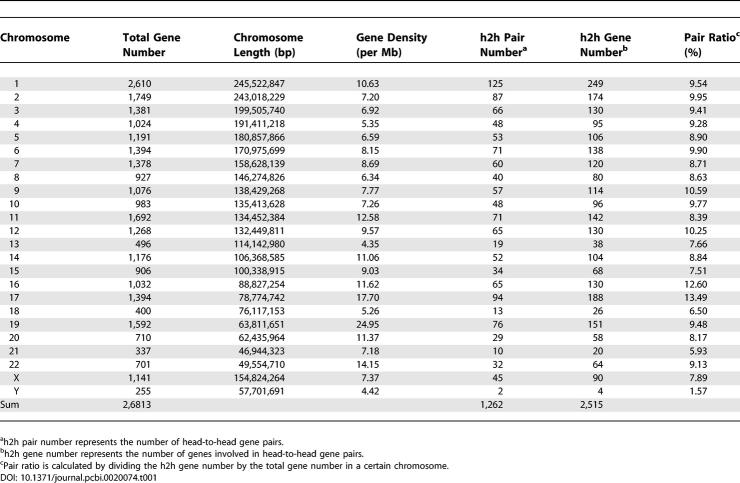
Distribution of Head-To-Head Gene Pairs on Each Chromosome

### Phylogenetic Analysis of Head-to-Head Gene Organization in Vertebrate Genomes

As there is a common profile of the distance distribution of head-to-head gene pairs for human, mouse, and rat, we attempted to determine if the head-to-head gene organization is conserved during vertebrate evolution. The *Fugu rubripes, Gallus gallus* (chicken), and human genomes were selected for this analysis. *Fugu* has the shortest known genome (approximately 365 Mb) of any vertebrate species, around one eighth of the size of the human genome [17]. The chicken has a genome of 1.2 Gb, approximately 40% of the size of the human genome and is the premier nonmammalian vertebrate model organism.

First, we identified orthologous gene pairs that remained consecutive with the same relative orientation in both human and *Fugu*. To detect orthologous genes in human and *Fugu,* 37,439 predicted *Fugu* peptides from the *Fugu* Genome Project were compared to 33,869 human peptides from Ensembl. According to the filtering criteria described by Aparicio et al. [17], 10,209 human-*Fugu* orthologous genes were determined. We mapped these genes to the human genome, and extracted 4,225 human consecutive pairs. Of these, 760 pairs (18.0%) were found to be consecutive with the same relative orientation in the *Fugu* genome, which represents gene pairs with conserved linkage between human and *Fugu* (Table 2). This proportion is comparable to Dahary et al.'s report [18].

**Table 2:**
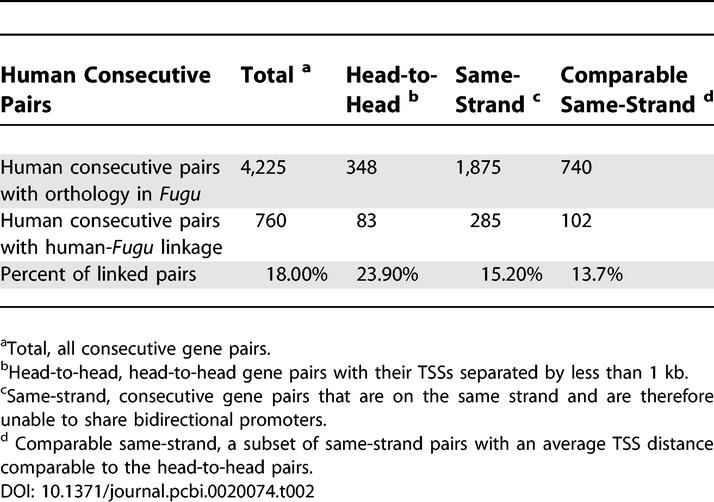
Conservation of Gene Pair Organization between Human and *Fugu*

Then we examined the conservation of head-to-head gene organization. Of the 4,225 human consecutive pairs with orthology in *Fugu,* 348 show the head-to-head organization, of which 83 (23.9%) keep the same organization in *Fugu* (Table 2). We used gene pairs that are consecutive and transcribed from the same strand in human as a control set (denoted “same-strand”). Only 15.2% (285 of 1,875) of the “same-strand” pairs in human have the same organization in *Fugu* (Table 2). These data indicate that head-to-head gene pairs tend to maintain their gene order significantly more than the background (total) and the control (same-strand) (*p*-value <5 × 10^−3^, by Fisher's exact test). Considering that the probability of rearrangement could depend on the distance between a pair of genes in the ancestral genome [19], we extracted 740 “same-strand” human pairs with an average distance comparable to that of the 348 head-to-head pairs to exclude the possibility that the observed rearrangement differences between head-to-head and “same-strand” pairs might be caused by differences in their original distance. Still, only 13.7% “same-strand” pairs had their gene order and orientation conserved (Table 2) (see Table S4 for detailed information).

It is known that the *Fugu* genome is highly compressed and the intergenic regions are very short compared to higher vertebrates [17, 20]. To check if head-to-head gene organization is conserved enough to influence the gene-distance expansion, we calculated genomic distances of gene pairs with human-*Fugu* linkage in human and *Fugu,* respectively. Due to the unavailability of full-length information for the *Fugu* genes, genomic distance was defined as the absolute value of the distance between protein-coding regions. For the entire group of 760 pairs with human-*Fugu* linkage, the average distance between a pair of genes in human was 8.90-fold larger than that in *Fugu,* which is in accordance with the difference between human and *Fugu* in genome size (Table 3). The “same-strand” group gives similar results. In contrast, only a 3.81-fold difference was observed for head-to-head gene pairs, with an average distance of 7.6 kb in human and 2.0 kb in *Fugu* (median, 1.3 kb and 1.6 kb, respectively) (Table 3). These results suggest a negative selection on the separation of head-to-head gene pairs, implying the ancestral existence of this gene organization.

**Table 3:**
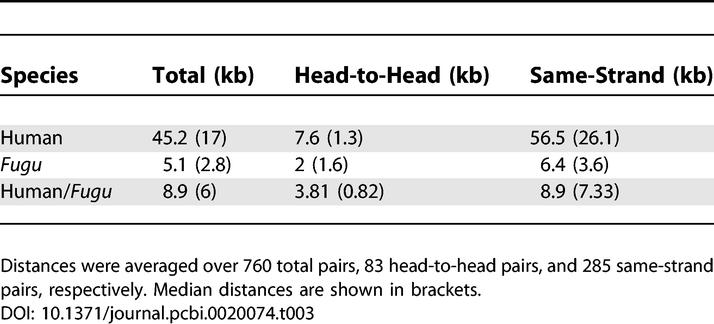
Genomic Distances of Gene Pairs with Human-*Fugu* Linkage

Furthermore, we analyzed the conservation of head-to-head gene organization between human and chicken genomes. By comparing 28,416 chicken peptides from Ensembl to 33,869 human peptides, 12,136 human-chicken orthologous genes were identified and mapped to human and chicken genomes. Then, 5,834 human consecutive pairs with orthology in chicken were extracted; of these, 3,490 pairs (59.8%) have conserved linkage between human and chicken (Table 4), which is much higher than between human and *Fugu* (18.0%) due to the closer phylogenetic relationship between human and chicken. Of the 5,834 human consecutive pairs, 384 show head-to-head organization, from which 264 (68.8%) keep this organization in chicken; in comparison, only 56.3% (1,491 of 2,646) of the control set, or “same-strand” pairs in human, are consecutive in the same strand in chicken (Table 4), indicating that head-to-head gene pairs significantly tend to maintain their gene order (*p*-value <5 × 10^−3^, by Fisher's exact test). For the same reason as above, we analyzed a group of 912 “same-strand” pairs that have an average distance comparable to that of the 384 head-to-head pairs and found that 60.5% (552 of 912) “same-strand” pairs had their gene order and orientation conserved, which is consistent with the background (59.8%) (see Table S5 for detailed information).

**Table 4:**
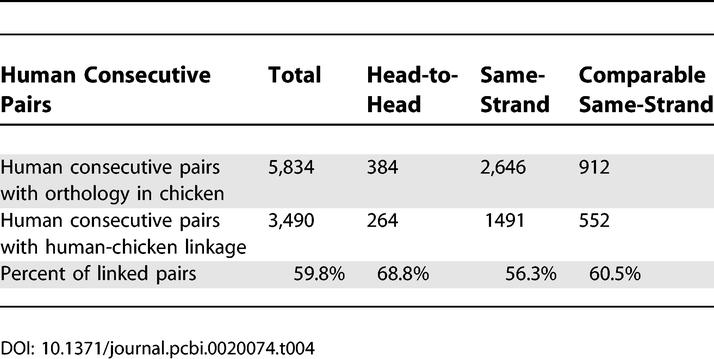
Conservation of Gene Pair Organization between Human and Chicken

We also calculated the genomic distance of each gene pair with human-chicken linkage in both human and chicken. For the entire group of 3,490 pairs, the average distance between genes was 2.89-fold larger in human than in chicken and similar to the “same-strand” group (2.93-fold), which is consistent with the difference between human and chicken in genome size (Table 5). In contrast, only a 1.59-fold difference was observed for head-to-head gene pairs (Table 5).

**Table 5:**
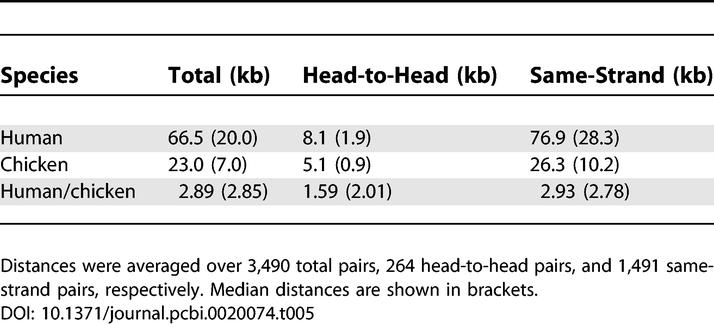
Genomic Distances of Gene Pairs with Human-Chicken Linkage

In addition, we calculated the genomic distances of gene pairs with human-chicken-*Fugu* linkage (Table S6). For the entire group of 325 pairs, the average distance between genes in human was 2.87-fold larger than in chicken and 9.97-fold larger than in *Fugu* (Table 6), which is comparable to the difference between human, chicken, and *Fugu* in genome size. The “same-strand” group again gives similar results. However, the average distance between head-to-head genes in human was only 1.25-fold larger than in chicken and 3.68-fold larger than in *Fugu* (Table 6). All of these data suggest the conservation of head-to-head gene organization during vertebrate evolution and thus the functional importance of this organization.

**Table 6:**
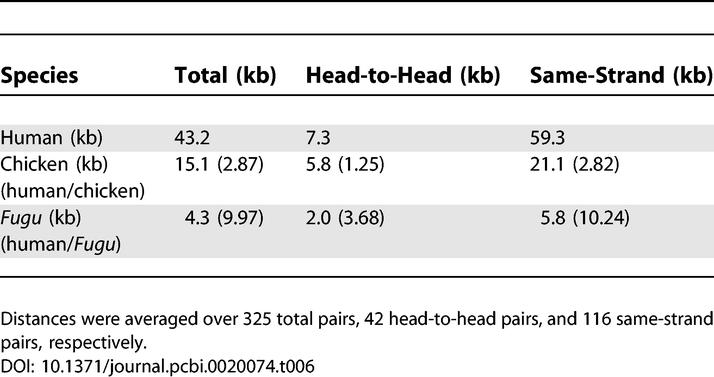
Average Genomic Distances of Gene Pairs with Human-Chicken-*Fugu* Linkage

### Expression Analysis of Human Head-to-Head Gene Pairs

**Figure 4:**
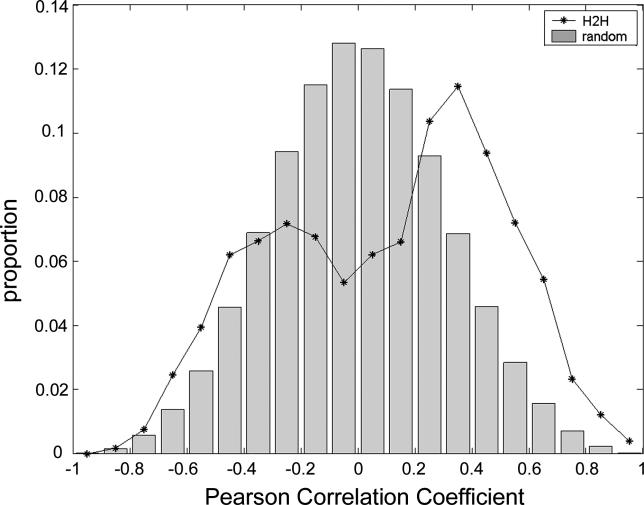
The Bimodal Distribution of the Expression Correlation between Head-to-Head Genes h2h, correlation distribution for head-to-head gene pairs, averaged over three distributions derived from three microarray datasets separately; random, correlation distribution for random pairs, also averaged over three random distributions from three microarray datasets.

The existence of a bidirectional promoter or potential shared *cis*-elements in a head-to-head gene pair raised the question about the transcriptional coregulation of the two involved genes. To investigate the transcription correlation between head-to-head genes, we mapped human head-to-head pairs to three human microarray datasets, E-MEXP-101, E-MEXP-230, and Jurkat (see Table S7 for original data), and obtained expression data for 369, 304, and 308 gene pairs in the three datasets, respectively. Then, we calculated the Pearson correlation coefficient of all gene pairs in each dataset independently (Table S8, “allH2H” sheet) and drew three distribution plots of correlation coefficient (Table S9, “allH2H” sheet). It was surprising that the expression correlations showed bimodal distributions with two peaks corresponding to positive and negative correlations, respectively, as this is apparently different from the previous report of a Gaussian distribution slightly shifted in the positive direction [2]. To exclude the possibility that a positive correlation of a gene pair in one experiment may cancel out a negative correlation in another experiment, we obtained an average distribution (Figure 4) by averaging the three distributions instead of averaging the correlation of each gene pair. It is noticeable that the average distribution is still a bimodal one with a large positive peak and a small negative peak (Figure 4).

Then we evaluated the significance of each correlation at *p* < 0.05 (Table S8, “allH2H” sheet). It was shown that among a total of 549 head-to-head pairs with available microarray data, 199 (36.2%) pairs show exclusively significant positive correlations, and 94 (17.1%) show exclusively significant negative correlations, according to at least one microarray dataset. Additionally, it is interesting that 49 pairs (8.9%) display positive or negative correlation depending on the condition of microarray experiments, indicating that alternative mechanisms may be involved in the transcriptional regulation of some bidirectional promoters. Considering that some of the 549 pairs have corresponding data in only one or two microarray datasets, but not all three datasets, the real proportion of alternative correlation could be higher than presented in this report. Overall, at least 62.3% of head-to-head genes show significant expression correlation. The negative correlation and alternative correlation were underestimated by previous studies [2].

### Functional Analysis of Human Head-to-Head Gene Pairs

All of the following functional analyses were based on Gene Ontology (GO) [21] annotations for head-to-head genes according to the association information provided by NCBI Gene Database (ftp://ftp.ncbi.nlm.nih.gov/gene). Of the 2,515 genes involved in the 1,262 human head-to-head pairs, 1,160, 1,019, and 1,075 genes were directly annotated by “biological process,” “molecular function,” and “cellular component” GO subsystems, respectively (Table S10, “all_DirectAnnotation” sheet). When both genes of a head-to-head pair are annotated by GO, the pair is denoted as an “annotated pair.” Of the 1,262 pairs, we obtained 267, 205, and 318 annotated pairs in the three subsystems respectively. As is mentioned in Materials and Methods, any direct annotation is generalized to all ancestor terms up to the root terms in our analyses, and “annotation” is meant as “general annotation” in the following context.

**Figure 5:**
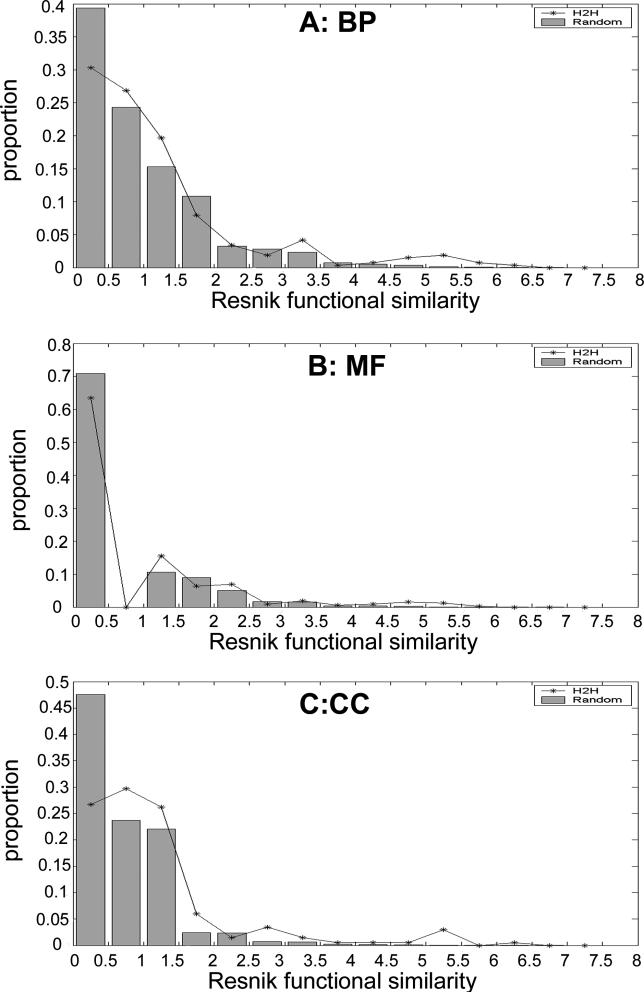
The Distribution of Functional Similarities for Head-to-Head Gene Pairs (A−C) GO subsystems “biological process,” “molecular function,” and “cellular component,” respectively.

In order to determine whether head-to-head genes statistically tend to perform similar functions, we evaluated functional similarities for annotated head-to-head pairs using the Resnik semantic measure. As is shown in Figure 5, the distribution of functional similarities for these pairs significantly shifts to larger values relative to those for random pairs, confirming the cofunction tendency observed in individual experiments. Since *p*-values by the Kolmogorov-Simirnov test are 0.0085 for “biological process,” 0.0126 for “molecular function,” and 4.2 × 10^−9^ for “cellular component,” respectively, head-to-head gene products are more likely to perform roles in the same cellular component, compared to the other two subsystems.

Then we set out to find out the GO terms which represent cofunctions of head-to-head pairs, or the functions whose associated genes tend to be organized in the head-to-head manner. Using a binomial probability model described in Materials and Methods, we obtained 22, eight, and 15 significant cofunctions (Table 7) in the “biological process,” “molecular function,” and “cellular component” subsystems, respectively, at a significance level of 0.01 (already adjusted for multiple testing error with the Bonferroni method). By merging the terms which point to closely related functions (see figures in the latter three sheets of Table S10 for the relationships of the cofunctions in each GO subsystem), we proposed that genes involved in functions including metabolism, chromosome organization and DNA packaging, anion transport, nucleic acid binding, catalytic activity, intracellular and organelle components, protein complex, collagen type IV, and so on, are more likely to be organized in the head-to-head configuration.

**Table 7:**
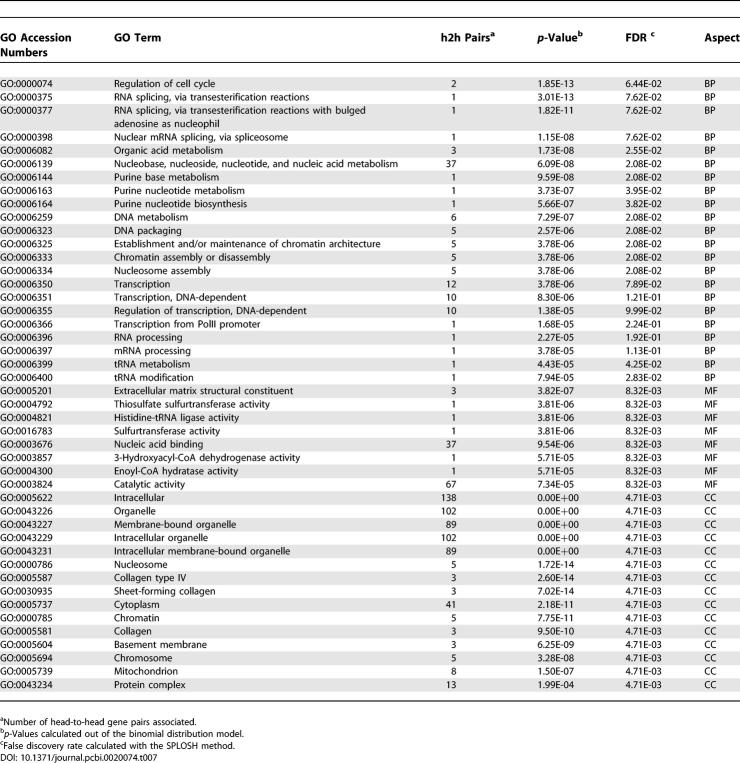
Significant Cofunctions Associated with Head-To-Head Gene Pairs in the “Biological Process” (BP), “Molecular Function” (MF), and “Cellular Component” (CC) Subsystems

To check the expression correlation between those head-to-head genes coding for similar functions, we extracted the expression correlation coefficients of the 282 pairs associated with the above 45 significant cofunctions (see Table S8, “cofunctionH2H,” sheet for details of expression correlation analysis; see the latter three sheets of Table S10 for association between cofunctions and gene pairs). Essentially, the expression correlation of head-to-head genes with cofunction is still characterized by bimodal distributions similar to the one shown in Figure 4 (Table S9 “cofunctionH2H” sheet). According to the Pearson correlation test, 80 (36.7%) and 45 (20.6%) pairs of the 218 pairs with available microarray data show significant positive and negative expression correlations, respectively, and 30 pairs (13.8%) display positive or negative correlation depending on the conditions of the microarray experiments. Overall, 71.1% of the cofunction pairs are significantly correlated, which is somewhat higher than that of background head-to-head pairs, 62.3%. It is interesting to note that the proportion of the third type (13.8%), alternative correlation, is higher than that for background (8.9%). These data suggest that the head-to-head genes coding for similar functions have stronger expression correlation - especially alternative correlation.

Here we focused on more specific GO terms rather than the terms with limited information content such as “metabolism,” even though they might have very small *p*-values. Five DNA packaging-related terms, including “nucleosome assembly,” “chromatin assembly or disassembly,” “establishment and/or maintenance of chromatin architecture,” “DNA packaging,” and “chromosome organization and biogenesis (sensu Eukaryota),” were ranked higher in the ascending list of *p*-values of the “biological process” terms. Also, the terms “nucleosome,” “chromatin,” and “chromosome” in the “cellular component” subsystem represent different aspects of similar functions. All of these nine terms coherently point to the following five head-to-head gene pairs, *HIST1H2BN*/*HIST1H2AK, HIST3H2BB*/*HIST3H2A, HIST1H2AH*/*HIST1H2BK, HIST2H2AC/HIST2H2BE,* and *HIST1H2BA/HIST1H2AA,* which are all histone coding genes (the first five entries in Table 8). Apart from these pairs, we also found 11 more histone-coding head-to-head pairs (the other 11 pairs in Table 8) in Table S1 according to the gene names and summaries provided by the NCBI Gene Database, which were not covered by the cofunction list (the latter three sheets of Table S10) because at least one member of a pair has not yet been annotated by the GO system. Taken together, the 16 pairs involve a total of 31 genes since *HIST1H2BF* could form two head-to-head pairs with overlapping genes *HIST1H2AD* and *HIST1H3D,* respectively. The 31 involved genes take 37% of a total of 83 genes located in the histone clusters. It is noticeable that all 16 pairs are organized in a nonoverlapping head-to-head manner, and most of them have very similar TSS distances. However, among the eight pairs with available microarray data, only one pair, *HIST1H2AC/HIST1H2BC*, shows positive expression correlations at *p* < 0.05. We could not exclude the possibility that the other pairs might have expression correlation under other experimental conditions.

**Table 8:**
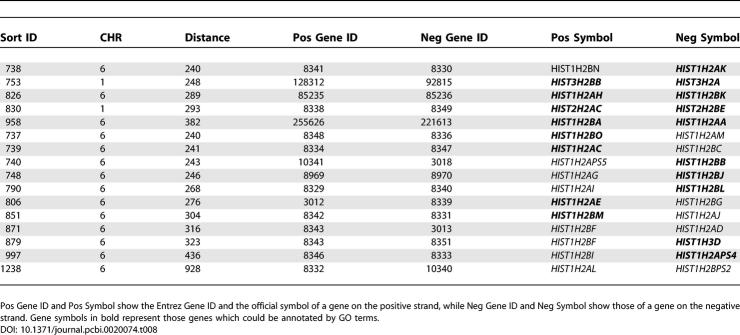
The Human Head-to-Head Gene Pairs Coding for Histone

We noticed that there are four collagen-related significant terms in the “cellular component” subsystem (Table 7), including “collagen type IV,” “sheet-forming collagen,” “collagen,” and “basement membrane,” coherently pointing to three head-to-head gene pairs, *COL4A2/COL4A1, COL4A3/COL4A4,* and *COL4A5/COL4A6*. They are all of the nonoverlapping type and located in Chromosomes 2, 13, and X, respectively. These three pairs were also annotated by several other significant cofunctions in the other two subsystems, such as “extracellular matrix structural constituent” in the “molecular function” subsystem and “inorganic anion transport,” “anion transport,” and “phosphate transport” in the “biological process” subsystem. Interestingly, the *COL4A2/COL4A1* pair and the *COL4A5/COL4A6* pair display significant positive expression correlations at *p* < 0.05; in contrast, *COL4A3/COL4A4* display a negative correlation.

## Discussion

Previous large-scale computational studies on human head-to-head gene pairs [2, 9], particularly by Trinklein et al. [2], dramatically advanced the recognition of the prevalence of this type of gene organization in the human genome. In the present work, we performed a systematic analysis of head-to-head gene organization, focusing on structural features, chromosomal distribution, evolutionary conservation, expression correlation, and functional association between involved genes.

### The Prevalence and the Structural Features of Head-to-Head Gene Pairs

In this study, 9.4% of the human genes were shown to be arranged in a head-to-head fashion, and this proportion is slightly smaller than the previous report of 11% based on cDNA alignment against genomic sequence [2]. With accession number conversion and matching, it was found that 594 (43.9%) of the 1,352 pairs identified by Trinklein et al. also appeared in our dataset, but in most cases the TSS distance calculated by Trinklein et al. is not consistent with our data (Table S11). Among the other 758 pairs in Trinklein et al.'s dataset, 129 have TSS distances larger than 1,000 bp according to the current data from NCBI Map Viewer; 596 cannot be handled due to lack of coordinate data or even lack of Entrez Gene IDs; and for 33 cases, two genes in one pair actually correspond to one gene (Table S11). Therefore, the inconsistency of these two datasets is at least partly attributed to the update of TSS coordinates during the accumulation of EST and mRNA evidence. We also checked the mouse and rat genomes and found that 8.2% and 4.4% of total genes, respectively, are head-to-head organized. It is well known that among the model species, human and mouse have the most abundant sequence information available. Taking dbEST as an example, there are 6,128,694 and 4,334,145 EST entries for human and mouse, respectively, in the release 072205 (July 22, 2005), but only 701,057 for rat. As a result, we believe that head-to-head genes in the rat genome might be under-estimated due to the limited mRNA and EST data.

TSS distance distributions of head-to-head genes in human, mouse, and rat genomes indicate that gene pairs with 1- to 400-bp TSS distance represent the majority of the total dataset with 62.36%, 64.15%, and 55.19% for human, mouse, and rat, respectively, and the largest group is the one with 101- to 200-bp distance. It should be noted that gene start sites in Map Viewer were regarded as TSS coordinates in this work as a compromise between accuracy and integrity for a genome-wide investigation, since DBTSS (http://dbtss.hgc.jp) presently provides exact TSS information of only 8,793 human genes [22] while Map Viewer provides genomic mapping data of 26,850 human genes based on extensive NCBI data. Due to the incomplete 5' UTR information of many genes, it is inevitable that we overestimate the TSS distances of some nonoverlapping head-to-head pairs and underestimate those of some overlapping pairs. Therefore, the peak column (101 to 200 bp) in the distance distribution (Figure 2) might actually move somewhat to the left or be much sharper. In fact, a peak of 200 to 300 bp was previously reported [2] based on the genomic data released before 2003. Considering the observation that the core promoter is always located in the 200-bp region upstream of a TSS, it is suggested that the peak column with 101- to 200-bp TSS distance is reasonable and might represent the most biologically relevant head-to-head gene pairs. Additionally, as is shown in Figure 2, the distance distribution of rat pairs showed a relatively flat profile, i.e., the column heights declined slowly away from the 101- to 200-bp distance, which might be also attributed to the incomplete 5' UTR information and thus the imprecise calculation of TSS distances.

### The Conservation and the Biological Relevance of Head-to-Head Gene Organization

The phylogenetic analysis of head-to-head gene organization among *Fugu,* chicken, and human suggests a negative selection on the separation of head-to-head gene pairs during vertebrate evolution, that is, the ancestral existence of these pairs. In fact, a considerable number of head-to-head pairs, for example, *COL4A1/COL4A2* [13], *DHFR/REP3* [10], *SURF-1/SURF-2* [11], *E14/ATM* [6], and *TK/KF* [12], have been found previously to be conserved among mammalian species. Since evolutionary conservation usually indicates functional importance, we proposed that the conservation of head-to-head gene organization has biological relevance to the function of the involved genes. This hypothesis was supported by the significant expression correlation and the functional association of head-to-head genes revealed in this paper, as well as that of Trinklein et al. [2].

The expression analysis indicated that a majority of human head-to-head pairs, 342 (62.3%) of 549 with available microarray data, show significant expression correlations. Among them, 58.2% are exclusively positively correlated, 27.4% are exclusively negatively correlated, and the other 14.3% are alternatively correlated depending on experimental conditions. Our studies suggest that the negative and alternative correlations were underestimated in previous studies. We attempted to examine the relationship between TSS distance and the degree of expression correlation using the Jonckheere-Terpstra test, but no significant relationship was observed (unpublished data). These findings implied, from a computational perspective, that a bidirectional promoter statistically tends to coordinately regulate the transcriptions of two involved genes in a TSS distance-unrelated manner and that the underlying mechanisms would be more complex than expected. Taking varicella-zoster virus *ORF28/ORF29* pair and mouse *TK/KF* pair as examples, the former pair can be expressed either coordinately or independently due to the existence of both shared regulatory element and distinct elements for each gene in the bidirectional promoter [20]. The latter one, *TK/KF,* is a typical antiregulated head-to-head pair [12]. The alternative activation of *TK* and *KF* genes seems to be based on their alternative response to the acetylation status of core histones associated with the bidirectional promoter. The transcriptions of *TK* and *KF* correlate with histone hyperacetylation and hypoacetylation, respectively. Until now, histone acetylation has been commonly thought to be a prerequisite for transcription initiation, and the *KF* gene is a rare example of a gene whose expression correlates with histone hypoacetylation [12]. It would be worth investigating if the correlation of hypoacetylation with transcriptional activation is more common than previously believed or if the mutual exclusive expression of head-to-head genes mainly depends on the mechanism involved in the *ORF28/29* example or other unknown mechanisms.

Our functional analysis indicated that head-to-head genes have the tendency to perform similar functions (Figure 5), which is reasonable considering the significant expression correlation of most pairs and the evolutionary conservation of this gene organization. It is consistent with the previous knowledge drawn from individual experiments that head-to-head arrangement helps genes perform functions in the same pathway [1, 13]. As is expected, head-to-head genes coding for similar functions have stronger expression correlation than the total pairs with microarray data. Besides the histone and collagen related pairs described in Results, we also observed that the “protein complex” term points to 13 pairs, and none of these pairs code for two subunits of one complex. Considering that seven of nine pairs are significantly correlated according to available microarray data, three positive, three negative, and one alternative (see Table S8, “InterestingCoFunctionH2H” sheet), we propose that the head-to-head organization might lead to some functional association of the two complexes in which the two genes of a pair are involved. In-depth research on these gene pairs may further reveal the biological relevance of their bidirectional gene configuration.

All these data suggested that the functional association or the biological relevance of head-to-head genes impose a restriction on gene order evolution and gene-distance expansion of vertebrate genomes. It is commonly assumed that higher eukaryotic genomes are loosely organized compared to simpler species. For vertebrates, the gene repertoires of human and *Fugu* are similar, although there has been a considerable scrambling of gene order and significant genome expansion with 8 times difference in size. However, the compact head-to-head gene organization seems to be a common architectural feature of vertebrate genomes. Combined with the previous studies on natural antisense transcripts [23, 24], the gene organization of eukaryotic genomes could be more complex than previously thought, and the transcriptional regulation based on gene organization could also be more prevalent and complicated. In fact, genes in the same region were found to be often coexpressed in the *Drosophila* and human genomes [25, 26]. Furthermore, synteny, gene regions keeping conserved across species, has been proposed to show expression correlation and functional association [27]. We believe the conservative head-to-head gene pairs contribute to the extensive distribution of synteny. A related work is still in progress.

It is well known that one of the major features of bacterial genomes is the arrangement of genes in operons, which facilitate gene coregulation and gene replication of heavily transcribed areas, and was thought to be an economic and ingenious strategy based on limited sequence source [28]. The head-to-head gene organization seems to use an exquisite strategy similar to operons in bacteria to achieve coordination between functionally related genes in eukaryotes. Remarkably, this organization enables both positive and negative correlation. Therefore, we feel that much more attention could be paid to research on eukaryotic gene organization and associated transcriptional regulation. In addition, the in-depth understanding of gene organization could help identify novel genes, in a similar manner to the identification of the *PACRG* gene linked to the *Parkin* gene via a bidirectional promoter [16]. At least the involvement of a predicted gene in a conserved gene organization will help confirm the prediction. We extracted 42 human head-to-head gene pairs with human-chicken-*Fugu* linkage and compared them to their ortholog pairs in chicken and *Fugu* (Table S6, “h2h” sheet). It was found that in ten cases, including *GNPTG/MGC24381, DNAI1/C9orf25, BYSL/USP49, ARPC4/TADA3L, PSMD13/SIRT3, MRPS11/MRPL46, NUDT1/FTSJ2, THEM2/TTRAP, ABCG8/ABCG5,* and *C17orf39/ATPAF2*, the evidence for both human genes are “reviewed” or “validated,” while the evidence for at least one gene in the orthologous pairs of chicken and *Fugu* is “novel.” Moreover, the genomic distances of human, chicken, and *Fugu* pairs are essentially comparable for the ten cases. As a result, the 20 genes involved in the ten pairs seem to be “real” in chicken and Fugu genomes and probably perform important conserved functions.

In conclusion, our genome-wide systematic analysis of head-to-head gene pairs on structural features, evolutionary conservation, and biological relevance greatly expanded our knowledge about this type of gene organization. It has been demonstrated that this highly conserved organization tends to subject functionally related genes to correlated transcriptional regulation. The existence of coexpression, mutually exclusive expression and alternative expression correlation suggests that the underlying mechanisms could be more exquisite and intricate than previously thought.

## Materials and Methods

### Data source.

Data sets of 26,850 human genes, 25,878 mouse genes, 21,977 rat genes, and their genomic mapping data were downloaded from the NCBI Map Viewer (ftp://ftp.ncbi.nih.gov/genomes/H_sapiens/maps/mapview/BUILD.35.1 ftp://ftp.ncbi.nih.gov/genomes/M_musculus/mapview/Build 34.1, and ftp://ftp.ncbi.nih.gov/genomes/R_norvegicus/mapview/Build 3.1). The exact TSS information for 9,550 human genes was downloaded from the DBTSS site version 5.0 (released in April 2005) (http://dbtss.hgc.jp).

A dataset of 33,869 known or novel human peptides was downloaded from Ensembl (NCBI35, May 2005; ftp://ftp.ensembl.org/pub/current_human/data/fasta/pep). A dataset of 37,439 predicted *Fugu* peptides and their mapping on the *Fugu* genome scaffolds was downloaded from the *Fugu* Genome Project (v3 assembly; ftp://ftp.jgi-psf.org/pub/JGI_data/Fugu). A dataset of 28,416 known or novel G. gallus (chicken) peptides and their mapping on the chicken genome scaffolds was downloaded from Ensembl (WASHUC1, May 2005; ftp://ftp.ensembl.org/pub/current_chicken/data/fasta/pep and ftp://ftp.ensembl.org/pub/current_chicken/data/mysql/gallus_gallus_core_31_1g respectively).

Two human gene expression datasets, indexed E-MEXP-101 and E-MEXP-230 respectively, were downloaded from the ArrayExpress database (http://www.ebi.ac.uk/arrayexpress). The third human gene expression dataset, denoted Jurkat dataset, was downloaded from the Stanford Microarray Database (SMD, http://genome-www5.stanford.edu/MicroArray/SMD). See below for details.

The associations of known human genes with GO terms were obtained from the NCBI Gene Database (ftp://ftp.ncbi.nlm.nih.gov/gene; March 2005). GO terms were downloaded from Gene Ontology Consortium (http://www.geneontology.org; January 2005).

### Identification of head-to-head gene pairs.

Since the precise determination of TSS of all human genes is far from complete, the gene start sites provided by NCBI Map Viewer were regarded as the coordinates of TSSs. Head-to-head gene pairs are defined as those divergently arranged gene pairs on opposite strands with TSSs not more than 1,000 bp apart according to Trinklein et al.'s study [2]. The dataset of head-to-head gene pairs was divided into two groups according to whether both transcripts overlap or not, with I and II representing overlapping and nonoverlapping gene pairs, respectively.

### Determination of gene pairs with conserved linkage between human, chicken, and *Fugu.*

One-to-one orthology relationships between human and *Fugu* genes, or human and chicken genes were determined using the method previously described [17]. Briefly, the *Fugu* (or chicken) proteins were compared to the human proteins and vice versa using BLASTP (BLOSUM62; E-value 1 × 10^−7^; identity 30%). The reciprocal best hits were taken as orthologs. Additionally, a similar BLASTP procedure was performed between chicken and *Fugu,* thus giving rise to a set of human-chicken-*Fugu* orthology relationships, where the orthologous proteins from every two species of the three form a reciprocal best hit.

Human consecutive pairs with orthology in *Fugu* or chicken were extracted using human genomic mapping data from NCBI Map Viewer. Note that Ensembl peptide ID was associated with NCBI Gene ID via Ensembl Martview. The gene pairs with conserved linkage between human and *Fugu,* or human and chicken were further identified according to the mapping data of *Fugu* or chicken genes.

Orientations (same-strand, opposite-strand) and distances between CDS coordinates of two genes involved in a consecutive pair were calculated also according to the above mapping data.

### Calculation and evaluation of the expression correlations between head-to-head genes.

We selected three human microarray datasets, E-MEXP-101, E-MEXP-230, and Jurkat (see Table S7 for original data), for expression analysis since each of them covers a large amount of genes.

The E-MEXP-101 dataset, from RKO colon carcinoma cells treated with either SIM2s control or antisense oligos in a time-course dependent manner, has 22,283 array spots and 32 arrays (Table S7, “E-MEXP-101” sheet). Of the 22,283 spots, 19,601 were mapped to 12,685 distinct NCBI Gene IDs according to the gene2unigene information provided by NCBI Gene Database (released in March 2005), and then 871 spot pairs, named as “head-to-head spot pairs,” were identified to represent 369 distinct head-to-head gene pairs.

The E-MEXP-230 dataset, the result of a time-course microarray assay designed to identify *FoxM1*-regulated genes [29], has 18,560 array spots and 14 arrays (Table S7, “E-MEXP-230” sheet). Of the 18,560 spots, 14,815 were mapped to 10,426 distinct gene symbols using relationship information between IMAGE clone ID and gene symbol provided by the SOURCE service (http://genome-www5.stanford.edu/cgi-bin/source/sourceSearch). Then, 538 head-to-head spot pairs representing 304 distinct gene pairs were identified.

The Jurkat dataset, aiming to compare expression profiles of Jurkat-derived T cell lines deficient for specific TCR signaling proteins to those of wild-type Jurkat T cells [30], has 37,632 rows and 21 columns (Table S7, “JurkatGEM” sheet). Of the 37,632 spots, 24,166 were mapped to 11,130 NCBI Gene IDs, and then, 1,318 head-to-head spot pairs representing 308 distinct gene pairs were identified.

Each array was normalized so that the log ratios had a mean of 0 and a standard deviation of 1. The Pearson correlation coefficients for the expression ratios of head-to-head gene pairs were calculated in each of the datasets separately. The correlations for 10,000 random spot pairs were also calculated within each of the datasets to evaluate the significance of head-to-head correlation.

In the case of multiple spot pairs mapping to an identical head-to-head gene pair, the maximum absolute value of the Pearson coefficient was chosen to represent the expression correlation, considering that the expression correlation tends to be overlooked by noise-contaminated, low-quality expression values. Using the maximum in this manner resembles the single linkage choice in hierarchical clustering, which has proved to be more robust against outliers than taking averages.

### Characterization of GO annotations for head-to-head and non–head-to-head genes.

GO (http://www.geneontology.org) is an authoritative gene functional categorization system that consists of three separate ontologies: “biological process,” “molecular function,” and “cellular component.” We obtained “direct annotations” of 14,801 known human genes according to the association table provided by NCBI Gene Database. According to the “true-path rule” (http://www.geneontology.org/GO.usage.shtml#truePathRule), if a gene is explicitly annotated to a lower-positioned, more specific term (direct annotation), then it is also implicitly annotated to the higher-positioned, more general terms that are on the paths from the directly annotated term to the root term of GO, by virtue of the parent-child relationship between these GO terms. So we generalized direct gene annotation to all ancestor terms up to the root terms, and defined them as “general annotation.” “Annotation” is meant as “general annotation” in the present paper.

### Evaluation of functional similarities between head-to-head genes.

The Resnik semantic measure, based on the information content of GO terms, was used to quantify the functional similarity between two genes in a head-to-head pair. *occ*_0_(*C_k_*) represents the number of human genes annotated by GO term *C_k_*. The Resnik probability *p*_0_(*C_k_*) is defined as 
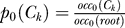
 . Note that a root term has a Resnik probability of 1, and a nonroot term has a Resnik probability less than 1. For genes *g*_1_ and *g*_2_ annotated by GO terms *C*_1_ and *C*_2_ respectively, the minimum subsumer term *C_ms_* is determined by Equation 1, where *S*(*C_1_,C_2_*) is a set of ancestor terms shared by both *C*_1_ and *C*_2_. Then the functional similarity between gene *g*_1_ and *g*_2_ is defined as Equation 2 shows.


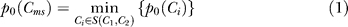



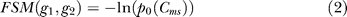


Besides the present 1,262 human head-to-head gene pairs, 50,000 random gene pairs were prepared, out of which 34,127, 29,044, and 40,286 pairs were annotated by “biological process,” “molecular function,” and “cellular component” GO subsystems, respectively. The functional similarities for these random pairs were calculated as a control. The similarities for head-to-head gene pairs and random pairs were analysed using the one-tailed Kolmogorov-Smirnov test [31] to find out whether the distribution of head-to-head pairs is significantly different from that of the control distribution and thus whether head-to-head genes tend to have similar functions.

### Determination of significant cofunctions associated with head-to-head gene pairs.

The expected probability of a random gene pair being annotated by *C_k_* is 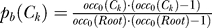 . In a certain GO subsystem, a particular minimum subsumer term *C_ms−i_* and all its ancestors are associated with the i-th head-to-head gene pair, and *occ_b_*(*C_k_*) is defined as the number of head-to-head pairs with GO term *C_k_* as their cofunction. Thus, under a binomial distribution model *B*(*n,p_b_*(*C_k_*)), the probability of *occ_b_*(*C_k_*) or more gene pairs randomly annotated by term *C_k_* is given by *p*(*C_k_*) = 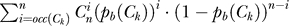 for a total of *n* annotated head-to-head pairs. This *p*-value can be used to evaluate the significance of cofunctions of head-to-head gene pairs. In the Table S10, we also estimated the false discovery rate (FDR) values accompanying these *p*-values using the SPLOSH method [32].

## Supporting Information

Figure S1 Chromosome Map of Human Head-to-Head Gene Pairs All of the mapped positions of the bidirectional gene pairs are represented schematically. The overlapping and nonoverlapping gene pairs are vertical lines below and above the horizontal line respectively
(46 KB JPG)

Table S1 Identification and Statistics of Human Head-to-Head (h2h) Pairs (260 KB XLS)

Table S2 Identification and Statistics of Mouse h2h Gene Pairs (752 KB XLS)

Table S3 Identification and Statistics of Rat h2h Gene Pairs (648 KB XLS)

Table S4 The Linkage of Consecutive Gene Pairs (csct), Head-to-Head Gene Pairs (h2h), and Same-Strand Consecutive Gene Pairs (ss) between Human and Fugu (1.4 MB XLS)

Table S5 The Linkage of csct, h2h, and ss Gene Pairs between Human and Chicken (2.0 MB XLS)

Table S6 The Linkage of csct, h2h, and ss Gene Pairs across Human, Chicken, and Fugu (138 KB XLS)

Table S7 Microarray Datasets Used in this Study. (12.9 MB ZIP)

Table S8 The Significance of the Expression Correlation for h2h Genes (837 KB XLS)

Table S9 The Distributions of h2h Gene Expression Correlations (179 KB XLS)

Table S10 The Associations of h2h Gene Pairs with Gene Ontology Terms (1.0 MB XLS)

Table S11 Comparison of Our Human h2h Pairs with a Previous Study (Trinlein et al.) (886 KB XLS)

## Abbreviations

bp: base pair
GO: Gene Ontology
NCBI: National Center for Biotechnology Information
TSS: transcription start site
