# Correction: Systematic Analysis of Head-to-Head Gene Organization: Evolutionary Conservation and Potential Biological Relevance

**DOI:** 10.1371/journal.pcbi.0020112

**Published:** 2006-08-25

**Authors:** Yuan-Yuan Li, Hui Yu, Zong-Ming Guo, Ting-Qing Guo, Kang Tu, Yi-Xue Li

.

In *PLoS Computational Biology*, volume 2, issue 7: DOI:  10.1371/journal.pcbi.0020074

The dataset links located in Materials and Methods are not active and should be replaced by the following:

ftp://ftp.ncbi.nih.gov/genomes/H_sapiens/maps/mapview/BUILD.35.1 should be http://rd.plos.org/10.1371_journal.pcbi.0020074_01

ftp://ftp.ncbi.nih.gov/genomes/M_musculus/mapview/Build should be http://rd.plos.org/10.1371_journal.pcbi.0020074_02

ftp://ftp.ncbi.nih.gov/genomes/R_norvegicus/mapview/Build should be http://rd.plos.org/10.1371_journal.pcbi.0020074_03

ftp://ftp.ensembl.org/pub/current_human/data/fasta/pep should be http://rd.plos.org/10.1371_journal.pcbi.0020074_04

ftp://ftp.ensembl.org/pub/current_chicken/data/fasta/pep should be http://rd.plos.org/10.1371_journal.pcbi.0020074_05

ftp://ftp.ensembl.org/pub/current_chicken/data/mysql/gallus_gallus_core_31_1g should be http://rd.plos.org/10.1371_journal.pcbi.0020074_06

